# The role of AI for MRI-analysis in multiple sclerosis—A brief overview

**DOI:** 10.3389/frai.2025.1478068

**Published:** 2025-04-08

**Authors:** Jean-Pierre R. Falet, Steven Nobile, Aliya Szpindel, Berardino Barile, Amar Kumar, Joshua Durso-Finley, Tal Arbel, Douglas L. Arnold

**Affiliations:** ^1^Department of Neurology and Neurosurgery, Montreal Neurological Institute, McGill University, Montreal, QC, Canada; ^2^Mila - Quebec AI Institute, Montreal, QC, Canada; ^3^Department of Electrical and Computer Engineering, Centre for Intelligent Machines, McGill University, Montreal, QC, Canada

**Keywords:** artificial intelligence, machine learning, magnetic resonance imaging, multiple sclerosis, precision medicine

## Abstract

Magnetic resonance imaging (MRI) has played a crucial role in the diagnosis, monitoring and treatment optimization of multiple sclerosis (MS). It is an essential component of current diagnostic criteria for its ability to non-invasively visualize both lesional and non-lesional pathology. Nevertheless, modern day usage of MRI in the clinic is limited by lengthy protocols, error-prone procedures for identifying disease markers (e.g., lesions), and the limited predictive value of existing imaging biomarkers for key disability outcomes. Recent advances in artificial intelligence (AI) have underscored the potential for AI to not only improve, but also transform how MRI is being used in MS. In this short review, we explore the role of AI in MS applications that span the entire life-cycle of an MRI image, from data collection, to lesion segmentation, detection, and volumetry, and finally to downstream clinical and scientific tasks. We conclude with a discussion on promising future directions.

## 1 Introduction

Multiple Sclerosis (MS) is a neuro-inflammatory disease of the central nervous system characterized by a wide spectrum of inflammatory and neurodegenerative changes (Compston and Coles, 2008), with clinical manifestations that vary greatly between individuals. Since the 1980s, magnetic resonance imaging (MRI) has been a cornerstone of MS diagnosis and management due to the ability to visualize demyelinating changes and axonal loss resulting from focal inflammation, using a combination of T2 and T1-weighted sequences (Hemond and Bakshi, 2018). The temporal evolution of lesions, which may initially enhance (Filippi et al., 2019), and subsequently expand, remain static, or decrease in size (Koopmans et al., 1989), can also be captured by MRI. A number of MRI biomarkers of MS diagnosis, prognosis, and treatment response, have also been described. These include T2-hyperintense white matter lesions, gadolinium-enhancing lesions, slowly enlarging lesions, paramagnetic rim lesions, cortical/deep gray matter lesions, and leptomeningeal enhancement (Filippi and Agosta, 2010; Filippi et al., 2020). Some of these biomarkers have been found to correlate strongly with key clinical outcomes. One example is the association between new/enlarging T2 lesions and clinical relapses (Rudick et al., 2006; Sormani et al., 2009; Sormani and Bruzzi, 2013).

Despite these advances, MRI-analysis continues to face problems that limit its potential (Maggi and Absinta, 2024). The longer acquisition times and higher field strengths required to obtain measurements of many recently studied imaging biomarkers introduces new headaches for resource-limited settings. At many clinical sites, the evaluation of MRI continues to be done manually, which is a lengthy, error-prone, and highly variable procedure (Bozsik et al., 2022; Altay et al., 2013). A strongly predictive imaging biomarker of disability progression, especially progression which is independent of relapse activity (Müller et al., 2023), has yet to be found (Filippi et al., 2020). At the therapeutic level, the influx of disease modifying therapies has significantly improved the ability to suppress lesion formation and relapse risk (Amin and Hersh, 2023), but targeting disability progression remains a major challenge. The use of MRI in predicting disease course and facilitating treatment selection is still a work in progress.

The rapid pace of progress in artificial intelligence (AI) has led to new opportunities for MRI-analysis in MS. In contrast to classical statistical methods which focus on acquiring knowledge about a population given data sampled from the same distribution, the field of AI has developed machine learning (ML) methods that focus on learning predictive patterns from a dataset with the aim of making predictions (generalizing) on new data (Bzdok, 2017; Bzdok et al., 2018). Some of this work provides a different perspective on—and a new set of solutions to—the current limitations of MRI-analysis.

When using the MRI modality as part of an AI system, practitioners often prefer to use a set of hand-crafted, image-derived features, which are based on well established image markers (e.g., T2 lesion counts, brain volume). These are typically scalars derived from the voxel-level data, either manually, or through a semi-/fully-automated process. The values for these hand-crafted features, which are easy to interpret, can be stored in tabular form, and used to train a model for a specific task using a variety of ML methods. Alternatively, the raw voxel-level data can be provided directly as an input to ML models. Some types of ML, in particular deep learning (DL), which uses deep artificial neural networks (LeCun et al., 2015), can make use of the high information content in voxel-level data to *learn* (automatically, without explicit guidance from a human expert) abstract, lower-dimensional features of the image that might not be captured by traditional hand-crafted, image-derived features (e.g., the texture of the white matter in a certain brain region). A specific type of deep neural network called the convolutional neural network (CNN) (LeCun et al., 1989; Li et al., 2022) has significantly advanced digital image processing by automatically learning features from images, sometimes leading to superior performance in tasks like image classification and object detection. The theoretical benefits resulting from ML on raw images come at the cost of greater computational and dataset requirements (Berisha et al., 2021), and generally require more expertise in model training. Traditional, hand-crafted features therefore remain valuable, especially in scenarios with limited data or specific constraints (Lin et al., 2020; Zare et al., 2018; O'Mahony et al., 2019).

**Figure 1:**
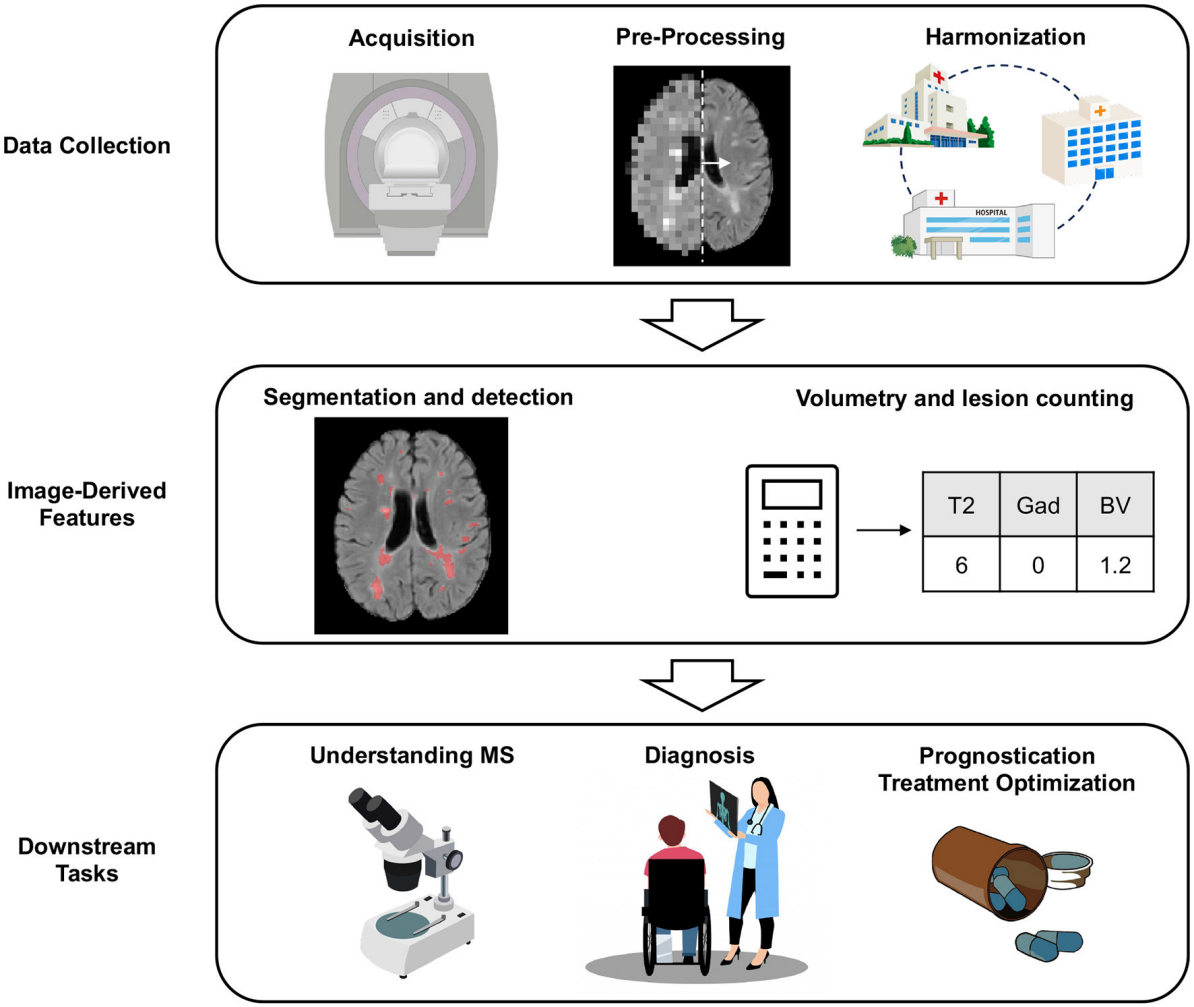
An overview of key areas in which AI is being used for MRI-analysis in MS.

This review aims to introduce the reader to key areas in which AI is transforming MRI-analysis in MS (see Figure 1 for an overview). Given the vastness of the literature on this topic, this review is meant to provide a high-level overview of selected areas that are of interest to the MS community, showcasing published work on MS-specific applications. As such, this does not represent a comprehensive review of the literature. Where possible, we refer the reader to more in depth, dedicated reviews, in specific sections. First, we will explore how AI can be used for data collection (Section 2), before discussing the traditional tasks of lesion segmentation, detection, and volumetry (Section 3). Finally, we will discuss downstream scientific and clinical tasks (Sections 4, 5, and 6). We end with a discussion on promising future directions (Section 7).

## 2 Acquisition, pre-processing, and harmonization

MRI has become essential for diagnosing MS and for monitoring it's evolution, primarily because of its higher sensitivity compared to clinical outcome measures of disease activity (McDonald et al., 1994). To reap the benefits of routine monitoring with MRI while minimizing the inconvenience for patients, caregivers, and resource utilization, many have turned to AI to improve the efficiency of MRI data collection. In this section, we will discuss three tasks pertaining to MRI collection: (1) acquiring the MRI images (acquisition), (2) processing the acquired images to improve their signal-to-noise ratio (pre-processing), and (3) transforming the pre-processed images from different scanners/sites to enable direct comparisons (harmonization).

Shortening the MRI acquisition time can be achieved by decreasing the number of sequences in the acquisition protocol, using generative models to synthesize the missing sequences. For example, Wei et al. (2019) showed that it is indeed possible to use a CNN to predict the FLAIR sequence from T1-weighted, T2-weighted, proton density, T1 spin-echo, and double inversion recovery (DIR) sequences. Others provided evidence to suggest that Generative Adversarial Networks [GANs, Goodfellow et al. (2014)] can synthesize DIR from the combination of T1 and T2/FLAIR (Finck et al., 2020, 2022), and T1 from T2-weighted FLAIR (Valencia et al., 2022). Although synthesis of gadolinium-enhanced T1-weighted sequences from low or non-contrast images is under-explored in MS, related work by Narayana et al. (2020) found that the presence of gadolinium-enhancing lesions can be predicted with moderate accuracy from non-contrast MRI.

Another strategy to speed data collection is to acquire lower resolution images, or images with a higher signal-to-noise ratio, and then use ML models in the post-processing phase to reconstruct higher-quality images. Various DL frameworks based on GANs and CNNs have been shown to produce higher-quality reconstructions that can improve lesion visualization and segmentation (Shaul et al., 2020; Zhao et al., 2019; Iwamura et al., 2023; Mani et al., 2021; Falvo et al., 2019). DL has also been used to optimize the more complex processing pipelines used for diffusion weighted imaging sequences (Golkov et al., 2016).

Finally, ML-based harmonization strategies can be used to address a frequently encountered problem in biomedical imaging research: small dataset sizes. Aggregating data from different data collection sites is complicated by the fact that each site may use different scanners and acquisition protocols, resulting in images that do not look alike. This is known to cause variability in tasks such as volume estimation (Clark et al., 2023; Bakshi et al., 2017). “Harmonization” is a solution to this problem that involves transforming the images so they all appear to come from the same distribution. Dewey et al. (2019) found benefits in the downstream task of brain volume estimation when images were first harmonized using DL. If direct visualization or comparisons between images from different datasets is not strictly necessary, one can also bypass the problem of harmonization by training models that are agnostic to the specific combination of sequences that is available for a particular patient (Havaei et al., 2016), or by searching for a set of hyperparameters that lead to comparable performance across a range of datasets (Gentile et al., 2023). It is worth noting that fake images can also be synthesized using DL to augment existing datasets. This is an open research problem and the magnitude of benefit probably depends on the context (Van Tulder and de Bruijne, 2015). Relatively little published research explores MRI generation specifically for MS datasets, but some authors have observed performance gains from augmentation with lesion-containing MRI images that are synthetically generated from the MRI images of healthy subjects (Salem et al., 2019; Basaran et al., 2022).

In summary, AI has shown promise in reducing the time taken to acquire and preprocess the MRI of MS patients, without significantly compromising the quality and utility of the MRI images. AI can also increase the ease with which data from different sources can be pooled together for further analysis, or for increasing the size of datasets which ML models use for training. Many of the methods that were reviewed in this section are at an early stage of development, and these tasks remain an active area of research.

## 3 Segmentation, lesion detection, and volumetry

Once a patient's MRI has been acquired and pre-processed, it is then ready to be used for clinical management and scientific research. Although the raw, voxel-level data can be fed directly as input to a ML model that is specifically trained for one of the downstream tasks described in Sections 4, 5, 6, there is often added value to taking an intermediate step consisting of identifying and quantifying established radiologic features in the images. These tasks include segmenting radiologic markers of MS, lesion detection, and the volumetric assessment of a variety of brain structures.

Current cross-sectional disease burden assessment typically consists of some variant on lesion volume, lesion count, and brain volume estimation. Monitoring of disease activity over time additionally calls for comparing volume estimates between time-points, and the detection of new or enlarging lesions. In most settings where radiologists and neurologists are responsible for performing these tasks, volume estimation is done qualitatively with high-level descriptors, while lesion detection is done using manual review of 2D slices. The process is lengthy, error-prone, and subject to significant inter- and intra-rater variability (Bozsik et al., 2022; Altay et al., 2013). For these reasons, there has been a growing appetite for at least partially automating these tasks using AI.

The segmentation of T2 lesions is one of the most well studied applications of ML in MS. The literature on automated MS lesion segmentation methods is vast, and methods range from classical ML to DL. We therefore refer the interested reader to several dedicated reviews for more details (García-Lorenzo et al., 2013; Danelakis et al., 2018; Spagnolo et al., 2023; Zeng et al., 2020; Doyle et al., 2018). There has been relatively less work on new (and/or enlarging) T2 lesion segmentation, but more emphasis has been placed on this task during recent challenges (Commowick et al., 2021). Beyond T2 hyper-intense lesions, DL has also been used to segment and detect imaging markers which are not currently integrated in most clinical settings. These include paramagnetic rim lesions (Barquero et al., 2020; Lou et al., 2021; Zhang et al., 2022), central vein sign on susceptibility-weighted images (Maggi et al., 2020), cortical lesions on 7T images (Rosa et al., 2022; La Rosa et al., 2020), gadolinium-enhancing lesions (Gaj et al., 2021; Karimaghaloo et al., 2010; Durso-Finley et al., 2020), and spinal cord lesions (Gros et al., 2019). The task of detecting lesions (including the detection of new lesions on follow-up images) has for the most part been studied in tandem with segmentation (Kamraoui et al., 2022; Salem et al., 2020; McKinley et al., 2020).

Although brain (parenchymal) volumetry has received less attention, DL has been used to segment the thalami of MS patients for the purpose of estimating its volume (Dwyer et al., 2021). DL methods have also been shown to perform well when compared to traditional methods for brain atrophy estimation (Zhan et al., 2023). Moreover, DL-based lesion-filling (or inpainting) has been shown to improve the performance of volumetric estimation methods that are usually sensitive to the presence of lesional tissue (Zhang et al., 2020; Clèrigues et al., 2023). Unfortunately, the large minimal detectable change in volume between clinically relevant intervals and the high inter-scanner variability still limit the utility of brain volume estimation in the clinic (Van Nederpelt et al., 2023). It is worth noting that a number of software packages for automated volumetric analysis and segmentation are available, and some already include DL methods (Billot et al., 2023).

Several challenges have been organized, in which groups compete for best performance on the same lesion segmentation task (either T2 lesion or new T2 lesion segmentation). These were hosted at the IEEE ISBI conference (Carass et al., 2017) and at MICCAI conferences (Styner et al., 2008; Commowick et al., 2018, 2021). In all cases, no model was found to be perfect, when evaluated on the basis of voxel-level segmentation metrics (under or over-segmentation) and lesion detection metrics (e.g., false positive rate), in comparison to the ground-truth segmentation obtained by human expert raters. Rather than indicative of a failure of ML for automatic segmentation, we argue that this finding should lead the community to rethink the way models are evaluated. In all challenges, performance was measured against the segmentation masks obtained from very few human experts, and on relatively small datasets of at most one hundred participants. Despite these challenge's best attempts to address the intra and inter-rater variability associated with the ground-truth lesion masks obtained from human experts (Bozsik et al., 2022; Altay et al., 2013), there remains no accepted consensus on what should constitute “ground truth”. Where should one draw the lesion border, given that lesional tissue manifests as a continuous spectrum of intensity on MRI? How do we differentiate an enlarging lesion from confluent new lesions? How do we know if hyperintensities smaller than 3 mm [which are typically disregarded by expert raters (Filippi et al., 2019) to avoid false positive detections], are pathologically significant or not? Without answers to all these questions, finding that DL methods disagree with human experts is arguably insufficient to determine if they are truly inferior. To address this issue, some have proposed explicitly modeling the “label-style” that might be associated with a certain dataset or group of expert-raters (Nichyporuk et al., 2022). Others have avoided the use of ground-truth lesion masks altogether by framing lesion segmentation as an unsupervised anomaly detection task (Behrendt et al., 2023; Castellano et al., 2022; Luo et al., 2023; Pinaya et al., 2022). Training on soft-labels (as opposed to binary labels) (Gros et al., 2021; Lemay et al., 2022) and probabilistic lesion counting (Schroeter et al., 2022) are yet other possible solutions. In recognition of the importance of the problem of model evaluation in the case of image analysis, a large international consortium has recently published recommendations for model evaluation (Maier-Hein et al., 2024; Reinke et al., 2024). Still, more work has to be done to obtain answers to the problems specific to MS lesion segmentation.

To conclude, segmentation, lesion detection, and volumetry, are some of the oldest and most studied ML application in MS. In many cases, they reach performances that are acceptable for many clinical and research settings. More work is needed to determine how best to evaluate automated segmentation frameworks.

## 4 Improving our understanding of MS

With an increasing number of datasets containing MRI images of MS patients, and the plethora of open questions in MS research, one may ask: could AI help us uncover novel markers of MS diagnosis, evolution, and treatment response? For years, patients with MS have been categorized into a binary classification system consisting of relapsing-remitting and progressive clinical phenotypes (Lublin and Reingold, 1996). It was later found that significant overlap exists in disease evolution across these subtypes, prompting the introduction of subtype-agnostic evolution-focused terminology such as “relapse-associated worsening (RAW)” and “progression independent of relapse-activity (PIRA)” (Lublin et al., 2022). The current most accepted perspective is that individual differences in disease course can be traced back to different combinations of inflammatory, neurodegenerative, and compensatory processes that lie along a continuous spectrum (Lassmann, 2019; Pitt et al., 2022; Vollmer et al., 2021).

This paradigm-shift, coupled with the fact that none of the existing MRI biomarkers have been particularly predictive of the key clinical outcome of disability progression (Filippi et al., 2020), has led researchers to search for alternative MRI-markers that could better explain the observed heterogeneity in disease evolution and treatment response. Notably, Eshaghi et al. (2021) and Pontillo et al. (2022) used an unsupervised ML algorithm called SuStaIn (Young, 2018) to identify disease subtypes characterized by distinct temporal progression patterns on MRI. Both groups found subtypes characterized by early cortical or deep gray matter atrophy, early signal changes in normal appearing white matter, and early T2 lesion accumulation. More work is needed to externally validate these subtypes and better understand their clinical correlates.

ML has also been used more directly to assist scientists in uncovering novel MRI markers. One strategy involves taking a pre-trained classifier (e.g., a model trained to predict MS diagnosis, or future disease activity) and producing “saliency-maps”. These allow researchers to visualize the features that are thought to be “important” according to the classifier; for example, features associated with a diagnosis of MS, poorer prognosis, or specific phenotypes. By using heatmaps generated using layer-wise relevance propagation, Eitel et al. (2019) found that a CNN classifier pre-trained to predict MS diagnosis focused on T2-lesions and their location, along with non-lesional or gray matter areas that included the thalamus. Storelli et al. (2022) produced heatmaps from a CNN that was trained to predict EDSS-worsening, and identified differences in periventricular regions, white matter lesions and the corpus callosum, for EDSS-worsened patients. Zhang et al. (2021) interrogated different heatmap-generating techniques to better understand crucial brain regions that could help distinguish MS phenotypes, finding that the abnormalities associated with SPMS were more extensive compared to RRMS, the latter involving primarily the occipital region and, to a lesser extent, the frontal region. Finally, Kumar et al. (2022) proposed to identify candidate biomarkers of future new/enlarging T2 lesions in an RRMS population through a process called counterfactual image synthesis; specifically, by predicting how a patient's MRI would look like if they had a different future outcome (a counterfactual), and by taking the difference between the real (factual) and counterfactual images, markers that are predictive of future outcomes (in this case, lesion activity) can be revealed.

AI can therefore be useful to better understand disease evolution and heterogeneity. While exciting, this work remains largely at the level of methodological development, and more translational research will be needed.

## 5 Diagnosis

It is imperative that an MS diagnosis be confirmed rapidly, and accurately, to ensure that patients receive the best possible care. MS is currently diagnosed according to the 2017 McDonald criteria, which combines historical, MRI, and laboratory data (Thompson et al., 2018). While significant efforts have been made to accelerate MS diagnosis, the heterogeneity of the disease and broad differential diagnosis still continues to put the clinician at risk of misdiagnoses, which can delay the initiation of an adequate treatment (Solomon et al., 2019; Brownlee and Solomon, 2021). Recent diagnostic criteria might provide increased sensitivity for the diagnosis, but at the cost of reduced specificity (Mescheriakova et al., 2018; Habek et al., 2018). In this section, we will discuss the use of AI for improving the accuracy and reliability of MS diagnosis. Note that there is some overlap with Section 3, since the detection of MS lesions on MRI is an important component of the diagnostic criteria (but not the only one). In the current section, the focus will be on the classification task of MS diagnosis, with the understanding that automated lesion segmentation and detection methods could be used upstream to provide image-derived features to an MS classifier.

Both classical ML and DL methods have been applied to the task of MS diagnosis, with MRI being the most common input modality for the classifier [we refer the reader to dedicated reviews on this topic for more details (Nabizadeh et al., 2022; Aslam et al., 2022; Shoeibi et al., 2021)]. Reported diagnostic sensitivity, and especially specificity, can be quite high [pooled sensitivity 92% (95%CI: 90%, 95%) and specificity 93% (95%CI: 90%, 96%), respectively, according to a recent meta-analysis (Nabizadeh et al., 2023)]. Even simple image-derived scalars such as the average of T1, T2*, and the total/myelin bound water content, have been found to be highly predictive (when used as input to train a supervised ML classifier) of an MS diagnosis (Neeb et al., 2019).

Differentiating MS from other diseases that can mimic it's presentation is also an important task in the clinic. Rocca et al. (2021) used a basic 3D-CNN with MRI as input to differentiate MS from neuromyelitis optica spectrum disorder (NMOSD), central nervous system vasculitis, and migraine, and found that the diagnostic accuracy exceeded that of human experts. Similarly, Kim et al. (2020) showed that MS could be differentiated from NMOSD using a 3D-CNN based on the ResNet architecture (He et al., 2016), as accurately as two neurologists. Huang et al. (2022) found that a transformer-based image classifier (Xu et al., 2021) could differentiate MS from NMOSD and myelin oligodendrocyte glycoprotein antibody disease as accurately as two neuroradiologists. MS could also be differentiated from hereditary diffuse leukodystrophy with spheroids using linear discriminant analysis (Mangeat et al., 2020), and from low grade tumors using MR-spectroscopy-derived features as input to a variety of ML models (Ekşi et al., 2021; Preul et al., 1996).

Overall, there is a growing amount of evidence supporting the use of AI in MS diagnosis.

## 6 Prognostication and treatment optimization

One of the main challenges for the clinician evaluating a patient with a new diagnosis of MS is to predict long-term prognosis (the evolution of the disease over time). The related task of treatment optimization (predicting which treatment will have the most beneficial effect) often depends on having an accurate prognosis. This begs the question: can AI do any better? Many early research efforts were focused on predicting the occurrence or timing of clinically-defined MS subtype transitions, using these as surrogate markers of poor prognosis. However, as discussed in Section 4, there has been a tendency to de-emphasize these subtypes in the diagnosis and management of MS. Prognostication tasks that we will focus on in this section therefore involve the prediction of the evolution of specific manifestations of the disease, which include radiologic activity (new/enlarging T2 lesions), relapses, disability accumulation, and patient-reported outcomes.

Prognostication with respect to disability outcomes turns out to be a very challenging task, even for AI (Seccia et al., 2021). When predicting disability progression from hand-crafted, image-derived tabular features, Pellegrini et al. (2020) found that a variety of classical ML models could achieve only modest predictive performance (C-index ≤ 0.65). Nonetheless, predictive performance can vary greatly depending on what features are used as input, on the model, and on the optimization procedure. With regards to the input, Zhao et al. (2017) found that classical ML methods performed better when adding image-derived features from a 1-year follow-up MRI visit to the set of inputs, which otherwise consisted of data recorded at a baseline visit. The benefit of longitudinal follow-up was also highlighted in work that used SuStaIn (Young, 2018) for unsupervised temporal modeling of imaging trajectories. Specifically, Pontillo et al. (2022) were able to identify a “deep-gray-matter-first” subtype that was associated with long-term cognitive impairment, and Eshaghi et al. (2021) could identify a “lesion-led” subtype that was associated with both confirmed disability progression and relapse rate. Using long term clinical (non-imaging) follow-up data has also been shown to lead to a considerable performance boost when predicting progression (De Brouwer et al., 2021). All this evidence suggests that ML on longer-term MRI data represents a promising, though challenging, research direction.

With regards to the model type, Zhao et al. (2020) found that ensembles of gradient-boosted trees such as XGBoost and LightGBM performed better than alternative ML methods when predicting 5-year EDSS worsening from logitudinal data collected over 2 years, with an area under the curve (AUC) ranging from 0.79 to 0.83. Interestingly, their feature importance analysis [and that of others (Law et al., 2019)] suggests that clinical disability metrics (which includes the EDSS) might be more predictive than tabular image-derived features for this particular task.

It is possible that voxel-level MRI data, which has been understudied for the task of predicting clinical prognosis, could harbor more predictive features of prognosis than traditional image-derived features. In support of this hypothesis, Storelli et al. (2022) were able to train a CNN to predict 2-year EDSS and SDMT worsening with 75.0% sensitivity, and 87.5% specificity. It is also possible that non-trivial implementation details, such as the inclusion of a T2-lesion mask along with the raw MRI as input, could further boost performance (Tousignant et al., 2019). These studies hint at DL's potential to improve upon tabular, hand-crafted, image-derived features (e.g., T2 lesion volume). In an attempt to elucidate the relative contribution of voxel-level data to predicting disability progression Zhang et al. (2023) studied a dataset of 300 MS patients, with a very large feature set spanning numerous MRI sequences, laboratory data, demographic information, disability scores, and unstructured clinical notes. Imaging, tabular data, and notes were encoded and fused using various neural network architectures, and used for predicting EDSS milestones 3-years later. While their best performing model made use of all three modalities (AUC 0.8380), a model trained without the MRI modality was only marginally worse (AUC 0.8078). Their study is limited by a small dataset size, with a comparatively large feature set, which could result in poor model optimization. More research is therefore needed to explore this important question, but this will require larger datasets, and additional methodological advances.

DL has also been used on radiologic markers of disease activity, which in certain cases are more sensitive to disease evolution than clinical measurements. A few studies have shown promising preliminary results in predicting the future appearance of new/enlarging T2 lesions from baseline MRI (Prabhakar et al., 2023; Durso-Finley et al., 2023, 2022). Tabular, hand-crafted image-derived features have also been used to classify a lesion as active or inactive (Peng et al., 2021). Similar to the task of predicting clinical prognosis (which focuses on predicting future disability-related outcomes), there remains the possibility that non-trivial methodological contributions may yield significant performance gains.

AI tools that aid in prognostication can be used for treatment optimization (for example, by favoring a more potent drug for a patient predicted to have highly active disease); however, it is also useful to consider the related task of estimating the “treatment effect” of a medication on the disease course. The most common treatment effect estimand that clinicians consider as part of treatment-related decisions is the *average* treatment effect, which typically is estimated using randomized clinical trials, and represents the average effect of a treatment on a population (compared to placebo or to a baseline drug). Some of the ML research cited in previous sections have presented results pertaining to treatment effect estimation. For example, the “lesion-led” subtype discovered by Eshaghi et al. (2021) appears to be specify a sub-group of individuals that experience a larger average treatment effect. Another line of work in causal ML aims to personalize treatment recommendations by predicting the treatment effect for a particular *individual* given their unique characteristics (Curth et al., 2024). For example, Durso-Finley et al. (2022) proposed a multi-headed CNN to predict the individual treatment effect of several treatments on new/enlarging T2-lesions, which used a person's MRI as input. Beyond treatment optimization, individual treatment effect estimation could also play a role in improving the statistical power of clinical trials by preferentially randomizing individuals who are predicted to benefit from an experimental therapy (Falet et al., 2022; Kanber et al., 2019).

In conclusion, although prognostication and treatment optimization remain challenging tasks, MRI-based ML research continues to improve upon previous baselines through diverse methodological innovations. Some models appear to identify subgroups of individuals that are more responsive to certain disease modifying therapies. These results are therefore paving the path toward precision medicine.

## 7 Discussion

In this review, we have presented several tasks where AI systems might already reliably outperform human experts in MS-specific applications. Indeed, a recent validation study by Barnett et al. (2023) provided evidence supporting the use of AI tools for lesion detection and volumetric analyses, in both clinical settings and research studies. We also discussed tasks which are hardly feasible without recent advances in DL, such as MRI sequence synthesis and automated biomarker discovery.

As the performance of AI tools continues to improve, we will arguably see increasing interest in trustworthiness, because these AI systems are expected to take part in high-risk human decision-making. Trust in AI systems is built in numerous ways, one of which is by giving them the ability to explain the rationale behind a model's predictions, resulting in “explainable” AI systems (Došilović et al., 2018). Additionally, users should be aware of the level of confidence that a model has in a particular prediction, and how much this reflects the actual errors that a model might make. This line of work, often referred to as “uncertainty” estimation (and the related problem of calibration), allows users to know when to trust a model's predictions (Gawlikowski et al., 2023). In addition, to trust that a model will behave well in practice, there should be a good understanding of how it will generalize to new data, and whether or not it will be robust to distribution shifts (for example, if there is a change in acquisition protocol). The field of causal machine learning (Sanchez et al., 2022), which models the data generative process using causal models, promises improved out-of-distribution generalization, and represents an active field of research. MS researchers have begun to address all three of these topics, specifically explainable methods (see examples in Section 4), probabilistic modeling for uncertainty estimation (Nair et al., 2020; Durso-Finley et al., 2023), and structural causal models of MRI image generation (Reinhold et al., 2021), but more work is needed to truly enable trustworthy AI-assisted MRI analysis in MS.

Looking forward, it seems clear that highly capable AI systems based on large foundation models (Brown et al., 2020; Devlin et al., 2018; Touvron et al., 2023; Ramesh et al., 2021) will have a major impact on biomedical imaging research, including in MS. Certain chat-bots based on large language models (LLMs) can now arguably pass the Turing test (Jannai et al., 2023), and score higher than the average human on medical exams (Achiam, 2023). LLMs are increasingly being used in medical applications (Agbavor and Liang, 2022; Patel and Lam, 2023; Singhal et al., 2023; Jiang et al., 2023), and multi-modal inputs (which includes biomedical imaging) are becoming more common (Moor et al., 2023). Although foundation models remain understudied in MS applications, interesting future directions include using foundation models to improve generalization from small MS-specific datasets, through in-context learning (Dong et al., 2024), or fine-tuning. That said, in order to reap all the benefits of foundation models for MS-specific applications, several open problems need to be solved. These include sub-par reasoning capabilities (Rae et al., 2021; McKenzie et al., 2023; Arkoudas, 2023) which could be dangerous in high-stakes environments such as healthcare (Richens et al., 2020; Fraser et al., 2018), broader concerns regarding AI safety (Bommasani et al., 2021; Anderljung et al., 2023; Urbina et al., 2022), and predictions that may be unacceptably skewed to the detriment of a particular group of people (Mehrabi et al., 2021). As more solutions to these problems are found, we can expect an increasing focus on large foundation models in the coming years, to help solve some of the most challenging tasks in MS MRI-analysis.
